# Periodontal disease influences osteoclastogenic bone markers in subjects with and without rheumatoid arthritis

**DOI:** 10.1371/journal.pone.0197235

**Published:** 2018-06-11

**Authors:** Jeneen Panezai, Ambereen Ghaffar, Mohammad Altamash, Per-Erik Engström, Anders Larsson

**Affiliations:** 1 Altamash Institute of Dental Medicine, Department of Periodontology, Karachi, Pakistan; 2 Karolinska Institutet, Department of Dental Medicine, Division of Periodontology, Huddinge, Sweden; 3 Habib Medical Centre, Rheumatology Clinic, Karachi, Pakistan; 4 Uppsala University, Department of Medical Sciences, Uppsala, Sweden; Katholieke Universiteit Leuven Rega Institute for Medical Research, BELGIUM

## Abstract

**Background:**

Periodontal disease (PD) and rheumatoid arthritis (RA) are bone pathologies mediated through immuno-inflammatory mechanisms. The aim of this study was to investigate the serum markers osteopontin (OPN), tumor necrosis factor receptors 1 (TNFR1) and 2 (TNFR2) receptor activator of nuclear factor‐kappa B ligand (RANKL) and RANKL/ osteoprotegerin (OPG) ratio and compare them in PD and RA groups.

**Materials & methods:**

RA (with PD = 19 and without PD = 19), PD (n = 38) and 14 healthy subjects underwent bleeding on probing (BOP) and probing pocket depth (PPD) measurement. PD was defined as PPD measuring ≥5mm registered in ≥3 sites. Marginal bone loss (MBL) for premolars and molars was measured on digital panoramic radiographs. Serum samples were collected from all subjects. OPN, TNFR1, TNFR2 and RANKL were measured by enzyme-linked immunosorbent assays (ELISAs). OPG was measured as part of a multiplex proximity extension assay (PEA).

**Results:**

OPN, TNFR1, TNFR2 and RANKL serum levels were the highest in the RA group with PD, while the RA group without PD were comparable to PD subjects only. The RANKL/OPG ratios were comparable between PD group and both RA groups with (p = 0.051) and without PD (p = 0.37). Serum RANKL levels were associated with MBL (p = 0.008) and PPD ≥ 5mm (p = 0.01).

**Conclusion:**

Peripheral osteoclastogenesis is a feature of periodontal disease with systemic levels of osteoclastogenic markers comparable to the effects observed in rheumatoid arthritis.

## Introduction

Interaction between the skeletal and immune systems in health is tightly regulated to maintain normal bone homeostasis. Dysregulation has dire consequences as seen in the abnormal host response and/or prolonged activation of the immune system leading to bone loss which is the hallmark in diseases such as rheumatoid arthritis (RA) and periodontal disease (PD) [1]. In a healthy state, osseous tissue turnover comprises of an episode of bone resorption followed by new bone formation. This process is referred to as coupling [2]. During inflammation, bone coupling is disturbed in favor of osteoclast-mediated bone resorption [3].

Osteopontin (OPN) has been implicated in a variety of disease states, where it mediates diverse cellular functions such as adhesion, migration, and survival of several different cell types, including regulating and propagating inflammatory responses of macrophages, T-cells and dendritic cells. OPN also functions as a Th1 cytokine, promoting cell-mediated immune responses, and plays a role in chronic inflammatory and autoimmune diseases [4]. Besides its function in inflammation, OPN is also a regulator of bone mineralization [5]. There is evidence that OPN not only helps in anchoring osteoclasts to bone surface but can also stimulate signal transduction in osteoclasts [6, 7]. Increased plasma OPN levels have been found in chronic inflammatory diseases such as Crohn’s disease and autoimmune diseases including rheumatoid arthritis, where plasma OPN is also associated with bone resorption markers. [8–11]. Tumor necrosis factor (TNF) has pleiotropic properties. TNF binds to two trans-membrane receptor molecules: TNFR1 (also sometimes referred to as p55) and TNFR2 (p75). TNFR1 and TNFR2 both exist as monomeric subunits on cell membranes as well as soluble forms of TNF receptors that are generated after proteolytic cleavage of the membrane forms. The biological interactions of TNF and TNFR induce inflammatory cytokines such as IL-6 [12]. TNF increases bone resorption, and reduces bone repair by exerting an inhibitory effect on osteoblast differentiation [13, 14]. In vitro, the proliferation of osteoblasts and the differentiation of osteoblast precursors, such as periodontal ligament cells, are significantly inhibited by TNF [6]. Another member of the TNF family, receptor activator of nuclear factor “kappa-light-chain-enhancer” of the activated B cells ligand (RANKL) is necessary for the formation, differentiation and activity of osteoclasts. Osteoprotegerin (OPG), along with its ligand RANKL, has been identified to play a role in the regulation of bone metabolism by mediating the signaling between osteoblast and osteoclast [15]. As a decoy receptor, the soluble OPG receptor binds to RANKL decreasing its availability for binding to RANK on osteoclast precursors, thereby negatively regulating osteoclastogenesis [16]. The phenomenon of spontaneous peripheral osteoclastogenesis has been reported to occur in cultures of peripheral blood mononuclear cells (PBMCs) from patients affected by diseases in which there is pronounced bone loss, including PD [17, 18, 19]. Underlying discrepancies in peripheral osteoclastogenesis might contribute to the pathogenesis of periodontal disease.

The aim of this study was to determine the levels of serological osteoclastogenic bone markers and RANKL/OPG ratios in PD and RA groups and compare the extent of systemic osteoclastogenic effects.

## Material and methods

### Patients

The study was performed at the Department of Periodontology, the Altamash Institute of Dental Medicine, Karachi, Pakistan. Informed consent was obtained from willing participants after giving information about the study. A detailed questionnaire was used to acquire information pertaining to hospitalization history and the presence of self-reported chronic diseases. Information regarding smoking habits, use of medication, oral hygiene status and past dental history was also recorded.

#### Rheumatoid arthritis (RA) group

A total of thirty eight RA patients with half of them suffering from periodontal disease (15 females and four males; age range: 27 to 60 years; mean ± SD: 50.4 ± 9.1 years) and the remaining half without periodontal disease (18 females and one male; age range: 21 to 70 years; mean ± SD: 41.8 ± 13 years) were diagnosed in accordance with the 2010 ACR / EULAR Rheumatoid Arthritis Classification Criteria and recruited from the Rheumatology Clinic in Habib Medical Centre, Karachi [20]. RA disease activity was measured with the Disease Activity Score 28 joint count (DAS28) by the rheumatologist (AG) [21]. All RA patients were being treated with disease modifying anti-rheumatic drugs (DMARDs), with or without concomitant non-steroidal anti-inflammatory drugs (NSAID) and/or steroids.

#### Periodontal disease (PD) group

Thirty eight subjects suffering from periodontal disease (26 females and 12 males, range: 25 to 64 years; mean ± SD: 47.4 ± 9.3 years) were included in the study. These subjects had no history or clinical diagnosis of RA, gout, or osteoarthritis.

#### Healthy (H) group

Another 14 healthy individuals attending the hospital (nine males and five females, range: 35 to 60 years; mean ± SD: 44.4 ± 6.6 years) were selected as controls. All controls had clinically healthy periodontium and no systemic disease.

Individuals presenting with osteoarthritis, gout, a history of treatment for periodontal disease during the last six months and / or treatment with antibiotics in the last three months were excluded.

### Blood collection

Four milliliters of fasting venous blood were collected from each participant using a BD Vacutainer™ plastic blood collection tubes without additives (4 mL). After allowing the blood to coagulate, the tubes were then centrifuged at 1790 x *g* for 10 minutes at room temperature. The serum was removed and transferred to 2ml storage tubes which were frozen and stored at -22°C until the time of assay.

### Clinical and radiographic examination

Bleeding on probing (BOP) was recorded, followed by measurement of probing pocket depth (PPD) in all teeth except for third molars. BOP was recorded as presence or absence of local bleeding within 30 seconds of probing. Periodontal disease was defined as three different sites or more with PPD of ≥5mm using a periodontal probe (Hu-Friedy manufacturing, Chicago, IL, USA). Measurements of PPD in the range of 3 - <5mm were defined as shallow pockets. The scores were recorded for four sites per tooth as a mean percentage for BOP and total number for shallow and deep pockets [20]. Additionally, digital panoramic radiographs were taken using a digital extra oral tomography machine (SironaOrthophos 3, Germany). The digital radiographs were viewed on a computer screen allowing for digital measurements to be made using SIDEXIS software. One pixel was equal to 0.09mm. Marginal bone loss (MBL) was defined as a measurement of the vertical distance from the cement-enamel junction (CEJ) to the most apical portion of the marginal bone. MBL was analyzed for premolars and molars (excluding third molars) in both arches. An average value for MBL per tooth was calculated after recording two subsequent readings of mesial and distal sides each on digital radiographs. Clinical and radiographic examinations were performed by the first author (JP).

### Immunoassay

Human Osteopontin (OPN), TNF R1, TNF R2 and RANKL were analyzed by commercial sandwich ELISAs (DY1433, DY225, DY726 and DY626, R&D Systems, Minneapolis, MN, USA). The assays utilized a monoclonal antibody specific for each peptide coated onto separate microtitre plates. Standards were assayed as duplicates and samples were assayed as singletons. They were pipetted into the wells and the peptides were bound to the immobilized antibodies. After washing, a biotinylated antibody was added. After another incubation and washing cycle, a streptavidine-HRP conjugate was added to the wells. After further incubation and washing, a substrate solution was added. The development was stopped and the absorbance was measured in a SpectraMax 250 (Molecular Devices, Sunnyvale, CA, USA). The peptide concentrations in the samples were determined by comparing the optical density of the sample with the standard curve. The coefficient of variation for the ELISAs were approximately 6%.

### OPG measurement using proximity extension assay (PEA)

Osteoprotegerin levels were analyzed as part of the Proseek Multiplex Inflammation I (Olink Bioscience, Uppsala, Sweden) at the Clinical Biomarkers facility, Science for Life Laboratory (Uppsala University, Sweden) according to the manufacturer's instructions [20]. The Proseek Multiplex analysis utilizes the Proximity Extension Assay (PEA) technology in which antibodies labelled with oligonucleotide probes, bind in pairs to their specific target protein. These antibody pairs are linked to each other via their unique DNA oligonucleotide sequences, which hybridize when in proximity. The DNA sequences are then extended and amplified by quantitative real-time PCR. The PEA data generated by Olink were reported in normalized protein expression (NPX) which is an arbitrary unit on Log2 scale where a larger number represents a higher protein level in the sample, with the background level at around zero. The calibrator curve for OPG is available at https://www.olink.com/products/inflammation/biomarkers/?biomarkerId=479.The assay protocols can be viewed using the link https://protocols.io/view/correlation-of-serum-cytokines-with-periodontal-di-jvkcn4w.

### Ethical approval

The study was approved by the ethics committee of the Altamash institute of Dental Medicine, Karachi, Pakistan (2012-09-26, 2016-09-30) and the Regional Ethical Review Board in Stockholm, Sweden (2016/296-31/1). All participants provided written informed consent prior to study enrollment according to the Declaration of Helsinki.

### Statistical methods

Data are reported as medians and interquartile ranges. Non-parametric statistics were used. Group wise comparison for analyzing the variables was performed using the Mann-Whitney test. Spearman rho coefficient was used to correlate between cytokines in the study groups and clinical parameters. To reduce the risk of false discoveries, the Benjamini-Hochberg method was used for adjusting p-values [22]. A p-value of 0.05 was considered statistically significant. For calculation of RANKL/OPG ratio, absolute RANKL concentrations were log transformed since NPX values for OPG cannot be converted to absolute protein concentrations, according to Olink (https://www.olink.com/products/inflammation/). The calculations were performed by use of SPSS version 21.0 (SPSS Inc, Chicago, IL, USA).

## Results

### Characteristics of study groups

Characteristics pertaining to the study subjects are presented in Tables 1, 2 and 3.

**Table 1:** Rheumatological characteristics of the rheumatoid arthritis (RA) group.

Characteristic	Rheumatoid Arthritis Group (N = 38)
Years since diagnosis	8.4 ± 6.23
DAS28	3.62 ± 0.99
IgM-RF (≥15 IU/mL) *	128.8 ± 149.8
ESR (mm/hr)*	53.7 ± 25.3
Anti-CCP (≥ 3 U/mL) *	256 ± 167.5

Values are shown as mean±SD

IU = international units

Anti-CCP values are expressed as arbitrary units (U/mL)

*Methodology [20].

**Table 2:** General characteristics for all study subjects. The total number of participating subjects was 90 with a mean age value of 46.4 years. Fifty percent of RA subjects had periodontal disease out of which 78.9% were females. The total number of subjects suffering from periodontal disease was 57 (with RA = 19, without RA = 38)*.

Total subjects (n)	90
Mean age distribution in years (range)	46.4 ± 10.1 (21–70)
Females (n, %)	64 (71)
Total subjects with RA (n)	38
Subjects with RA and PD (n, %)	19 (50)
Subjects with RA and without PD (n, %)	19 (50)
Total subjects with PD only (n)	38
Healthy Controls (n)	14

*Previousy published as Table 2 in vol.12 [20].

**Table 3:** Rheumatologic characteristics of RA subjects with and without PD*.

Characteristic	RA without PD (n = 19)	RA with PD (n = 19)
Years since diagnosis	8.7	8.1
IgM-RF (≥30 IU/mL)	117.5	168.7
ESR (mm/hr)	49.8	54.8
Anti-CCP (≥ 3 U/mL)	251.7	286.1

*Previousy published as Table 3 in vol.12 [20].

Characteristics comprising of disease duration since the time of diagnosis, IgM-RF, ESR and anti-CCP values are shown for RA subjects with and without PD in Table 3. DAS28 score for RA subjects with PD was 4.02 and 3.51 for those without PD.

### Clinical parameters

Comparison of clinical and radiographic parameters between RA (with and without PD), PD and healthy groups are presented in Table 4 [20]. Mean values for BOP, PPD≥5mm and MBL were found to be the highest in the PD group. There was no difference in the number of pockets measuring 3-<5mm between the RA subjects (with and without PD) and PD group. Also, no difference was found in the number of teeth amongst all four groups.

**Table 4:** Comparison of clinical and radiographic parameters between RA (with and without PD), PD and control groups^a^.

	RA without PD (n = 19)	RA with PD (n = 19)	PD (n = 38)	Controls (n = 14)
**BOP%**	42.6 ± 31‡	37 ± 32	76.2 ± 27*‡§	27.2 ± 29.4
**PPD 3- <5mm**	55.5 ± 16.2§	43.5 ± 17.9**	49.2 ± 17.4§	26.9 ± 14.1*
**PPD≥ 5mm**	0.32 ± 0.74‡	18.2 ± 15.2§	33.4 ± 14.1*‡§	0.4 ± 0.5
**Number of Teeth**	26.8 ± 2.9	25.5 ± 4.1	24.8 ± 4.3	26.1 ± 4.7
**Mandibular MBL mm**	2.9 ± 1	3.8 ± 2.2	5.4 ± 2.6*‡§	3.3 ± 0.7

Differences in the means ± SD of clinical parameters were tested using one-way ANOVA. For multiple comparisons, post-hoc Tukey HSD test was performed.

* compared to RA without PD. P<0.01

‡ compared RA with PD. P<0.01

§ compared to healthy controls. P<0.01

** compared to healthy controls. P<0.05

BOP = Bleeding on probing, PPD = Probing pocket depth, MBL = marginal bone loss

RA = rheumatoid arthritis PD = periodontal disease

a Previously published as Table 4 in vol.12 [20].

### Serum levels of OPN, RANKL, TNFR1 and TNFR2

**Fig 1:**
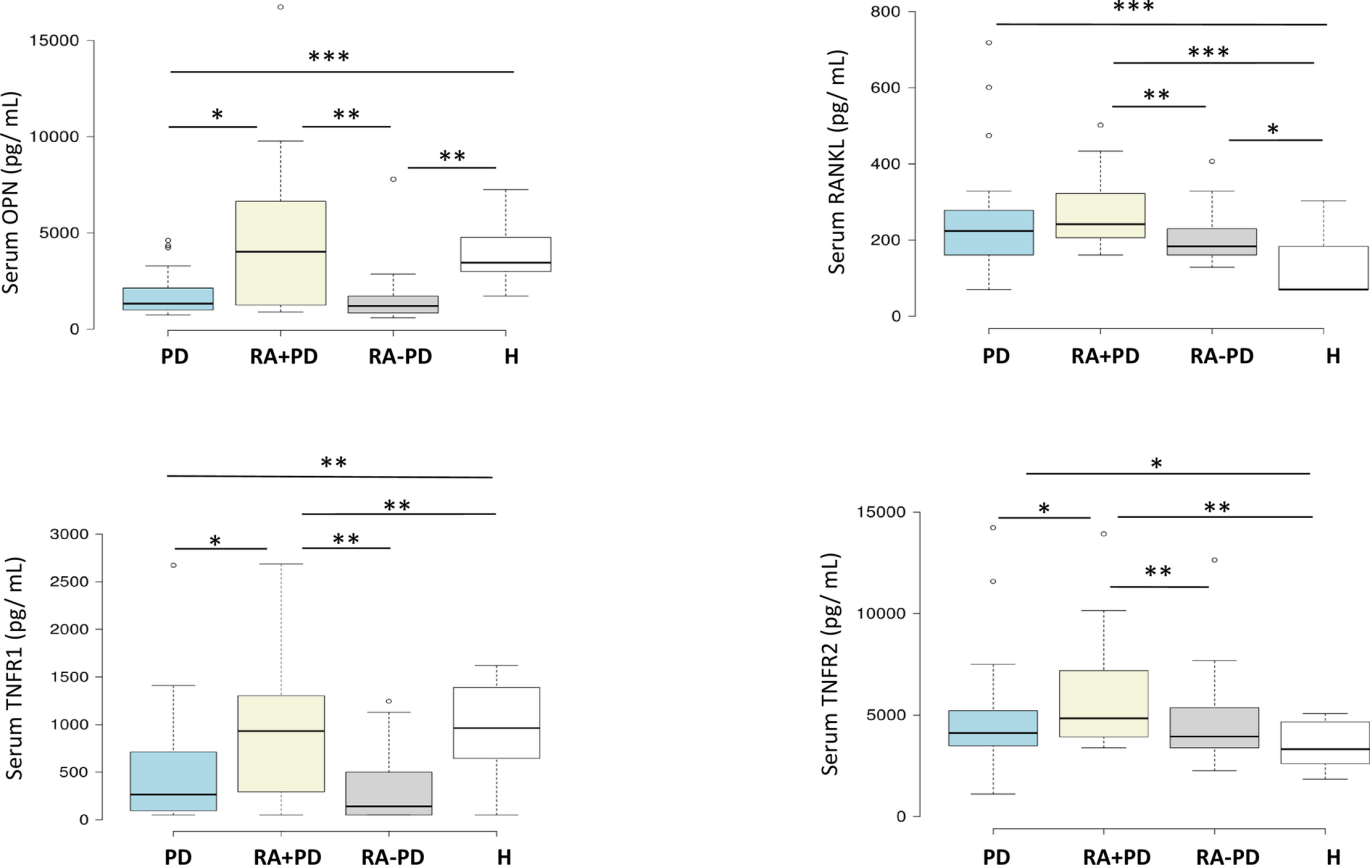
Boxplots representing the median (horizontal line), interquartile range (upper and lower) and upper and lower limits and outliers (dots) of osteopontin, RANKL, TNFR1 and TNFR2 serum levels in periodontal disease (PD, n = 38), rheumatoid arthritis with PD (RA+PD, n = 19), rheumatoid arthritis without PD (RA-PD, n = 19) and healthy (H, n = 14) groups. * p<0.05 ** p<0.01 *** p<0.001.

Serum concentrations of OPN, TNFR1, TNFR2 and RANKL for four groups RA with PD, RA without PD, PD and H groups are presented in Fig 1. There were no statistical differences between the PD and RA without PD groups for all four markers. However, all markers were increased in RA subjects with PD when compared to those without PD. Levels of RANKL were comparable between PD and RA subjects (with and without PD). Osteopontin and TNFR2 levels were the highest in the RA with PD group.

### Correlations between clinical and serological data

Serum RANKL was associated with number of PPD≥5mm (r = 0.28, p = 0.01) and MBL (r = 0.29. p = 0.008). OPN was inversely associated with number of PPD 3 - <5mm (r = -0.31, p = 0.03) whereas TNFR1 inversely correlated with PPD ≥ 5mm (r = -0.32, p = 0.003). Results are presented in Table 5.

**Table 5:** Correlation of RANKL, OPN, TNFR1 and TNFR2 serum levels with periodontal characteristics* of all subjects via estimation of the Spearman rank correlation coefficient (r). The Benjamini and Hochberg procedure was used for multiple testing (adjusted p-values).

	Spearman's rho (r)	p-value	Adjusted p-value
**RANKL**			
BOP	0.07	0.56	
PPD 3-<5mm	-0.14	0.22	
PPD≥5mm	**0.28**	**0.010**	0.013
MBL	**0.29**	**0.008**	0.009
**OPN**			
BOP	-0.18	0.09	
PPD 3-<5mm	-**0.31**	**0.003**	0.006
PPD≥5mm	-0.02	0.85	
MBL	0.13	0.27	
**TNFR1**			
BOP	0.12	0.25	
PPD 3-<5mm	-0.09	0.38	
PPD≥5mm	**-0.32**	**0.003**	0.003
MBL	-0.24	0.032	0.019
**TNFR2**			
BOP	-0.23	0.028	0.016
PPD 3-<5mm	0.01	0.913	
PPD≥5mm	0.17	0.115	
MBL	0.23	0.05	0.022

*BOP = Bleeding on probing; PPD = Periodontal probing depth; MBL = Marginal bone loss

### Group wise correlation between clinical and serological data

A group wise comparison showed that the RA with PD group had the most correlations between serum analytes and clinical parameters. RANKL, OPN and TNFR1 correlated negatively with BOP and number of shallow pockets (PPD3-<5mm) in this group. Results are shown in Table 6. In the PD group, only TNFR1 was inversely associated with MBL whereas in the RA-PD group, no significant correlations were found.

**Table 6:** Group wise correlation of RANKL, Osteopontin, TNFR1 and TNFR2 serum levels with periodontal characteristics* in rheumatoid arthritis (RA) with PD, without PD and periodontal disease (PD) groups.

RA- PD Group (n = 19)			RA + PD Group (n = 19)			PD Group (n = 38)		
	*r*	*P-value*:		*r*	*P-value*:		*r*	*P-value*:
**RANKL**			**RANKL**			**RANKL**		
BOP	-0.03	0.91	BOP	**-0.46**	**0.05**	BOP	0.07	0.67
PPD 3<5mm	-0.21	0.38	PPD 3<5mm	**-0.50**	**0.03**	PPD 3<5mm	0.06	0.74
PPD≥5mm	NA^**^		PPD≥5mm	0.15	0.55	PPD≥5mm	0.02	0.91
Total MBL	-0.12	0.633	Total MBL	-0.21	0.40	MBL	-0.27	0.10
**Osteopontin**			**Osteopontin**			**Osteopontin**		
BOP	-0.34	0.15	BOP	**-0.62**	**0.004**	BOP	-0.21	0.21
PPD 3<5mm	0.08	0.73	PPD 3<5mm	**-0.57**	**0.01**	PPD 3<5mm	0.13	0.43
PPD≥5mm	NA		PPD≥5mm	-0.05	0.84	PPD≥5mm	0.13	0.43
MBL	0.19	0.43	MBL	-0.20	0.42	MBL	-0.32	0.051
**TNFR1**			**TNFR1**			**TNFR1**		
BOP	-0.35	0.14	BOP	**-0.51**	**0.02**	BOP	-0.08	0.65
PPD 3<5mm	-0.30	0.22	PPD 3<5mm	**-0.62**	**0.00**	PPD 3<5mm	0.11	0.50
PPD≥5mm	NA		PPD≥5mm	0.27	0.26	PPD≥5mm	0.18	0.29
MBL	0.17	0.49	MBL	0.09	0.71	MBL	**-0.45**	**0.0044**
**TNFR2**			**TNFR2**			**TNFR2**		
BOP	-0.30	0.21	BOP	-0.05	0.84	BOP	-0.11	0.51
PPD 3<5mm	-0.10	0.67	PPD 3<5mm	-0.41	0.08	PPD 3<5mm	0.06	0.72
PPD≥5mm	NA		PPD≥5mm	0.03	0.92	PPD≥5mm	-0.14	0.40
MBL	0.44	0.06	MBL	0.01	0.96	MBL	-0.21	0.21

*BOP = Bleeding on probing; PPD = Periodontal probing depth; MBL = Marginal bone loss

**NA: not applicable as these subjects did not meet the criteria of having 3 or more pockets measuring ≥ 5mm

### OPG levels and RANKL/OPG ratio

**Fig 2:**
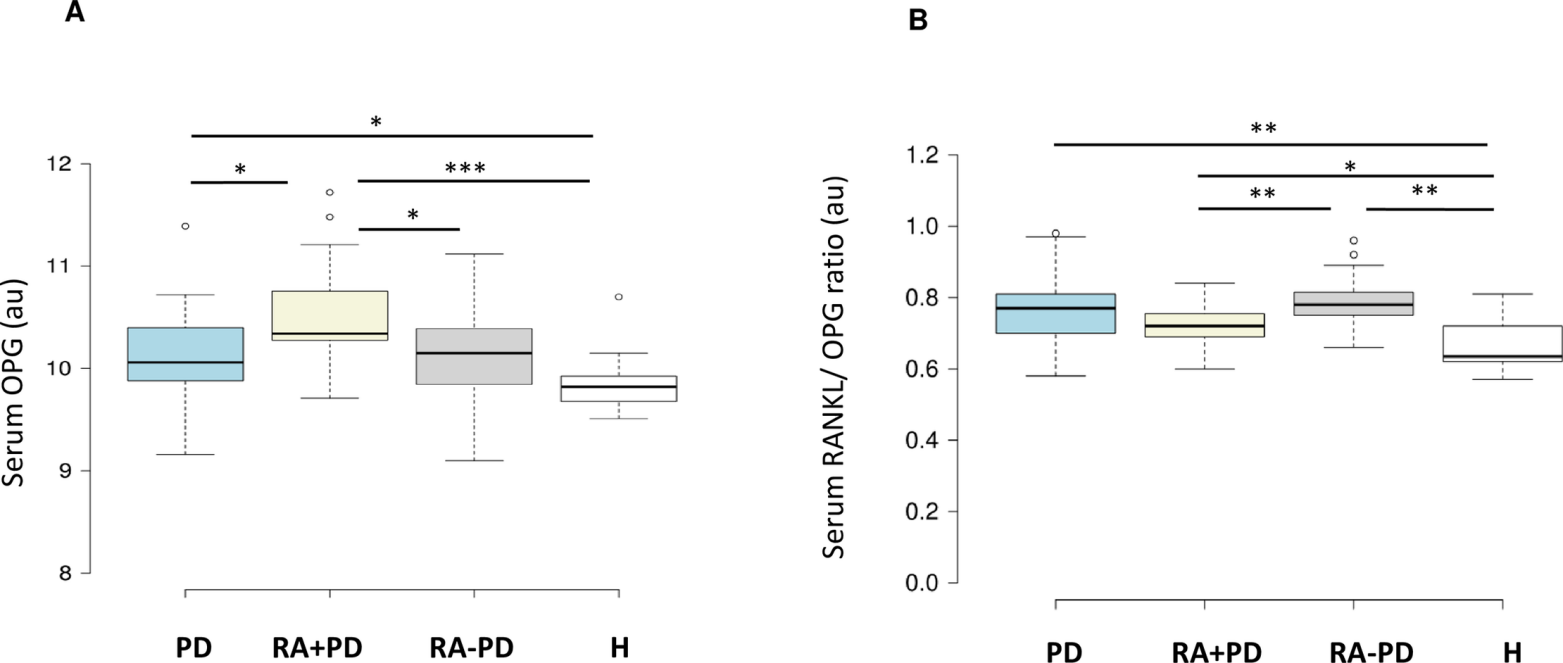
**Comparison of OPG levels and RANKL/ OPG ratio. Data are shown as boxplots representing the median (horizontal line), interquartile range, upper and lower limits and outliers in (A) serum OPG levels in periodontal disease (PD, n = 38), rheumatoid arthritis with PD (RA+PD, n = 19), rheumatoid arthritis without PD (RA-PD, n = 19), and healthy (H, n = 14) groups. (B) Serum RANKL/ OPG ratios.** * p<0.05 ** p<0.01 *** p<0.001 au = arbitrary units.

The serum OPG levels for four groups are shown in Fig 2A. Highest levels were seen in RA with PD subjects as compared to the remaining three groups. There was no difference between the PD and RA without PD groups. Serum RANKL/OPG ratios are shown in Fig 2B. The ratios were comparable between PD subjects and both RA groups. However, the ratio was higher in RA without PD as compared to those with PD. All groups showed higher ratios when compared to healthy individuals.

### RANKL and MBL

**Fig 3:**
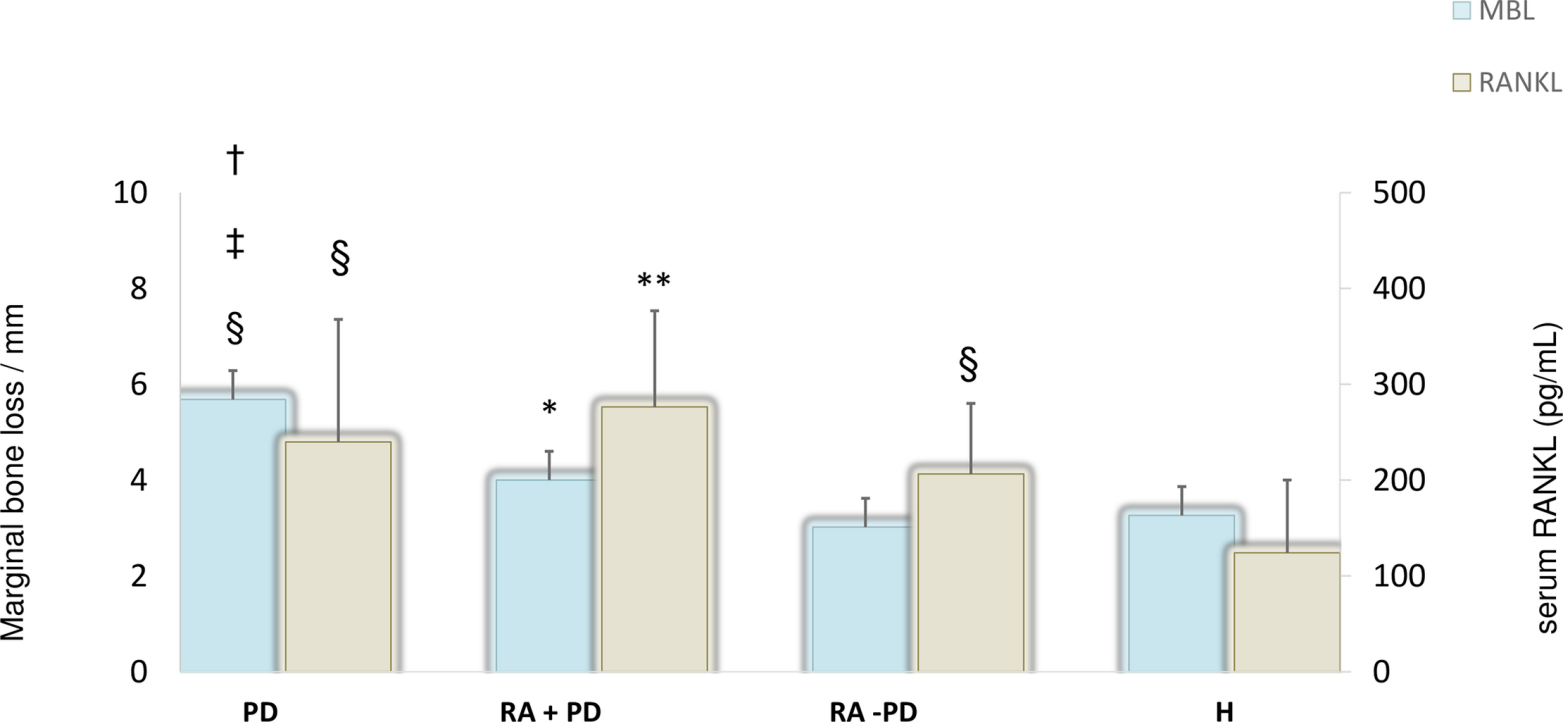
Comparison of mean values of marginal bone loss (MBL) and serum RANKL levels in periodontal disease (PD, n = 38), rheumatoid arthritis with PD (RA+PD, n = 19), rheumatoid arthritis without PD (RA-PD, n = 19), and healthy (H, n = 14) groups. ᵻ p<0.01 compared to RA+PD ⱡ p< 0.01 compared to RA-PD * p<0.05 compared to RA-PD § p < 0.01 compared to H ** p<0.001 compared to H.

The mean RANKL levels and their variation with mean MBL values for the study groups are shown in Fig 3. The MBL values varied in accordance with serum RANKL in PD and H groups whereas both the RA groups showed higher RANKL for lower MBL values.

### Correlation between analytes

**Fig 4:**
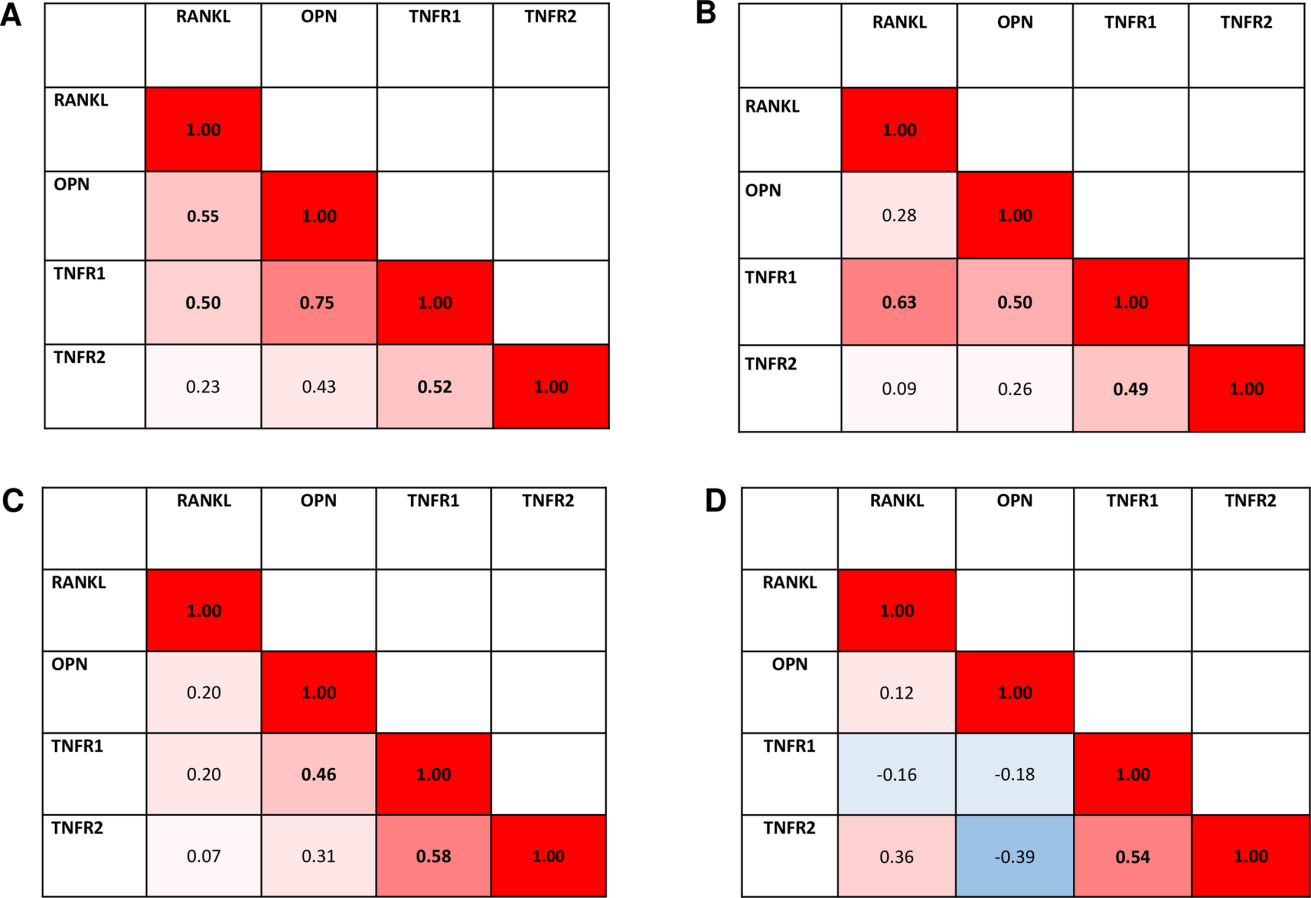
Spearman rho coefficients between analytes. **(A) Rheumatoid arthritis with PD (RA+PD, n = 19), (B) rheumatoid arthritis without PD (RA-PD, n = 19), (C) periodontal disease (PD, n = 38) and (D) healthy (H, n = 14) groups. Red areas indicate positive correlation between analytes, and blue areas indicate negative correlation between analytes.** Significant associations (p<0.05) are shown in bold.

Correlation between analytes was done pairwise for all four study groups. Results are shown in Fig 4. The RA with PD group showed the most correlations between pairs of analytes. Correlations were significant for four out of six pairs showing the strongest correlation between OPN and TNFR1 (r = 0.75). RA subjects without PD had three out of six possible correlations with the strongest correlation between RANKL and TNFR1 (r = 0.63). TNFR1 and TNFR2 were significantly associated in all four groups, the strongest in PD (r = 0.58).

## Discussion

Pathogenic bone loss is more attributed to an increase in bone resorption rather than reduced bone formation for which osteoclasts are considered to be the responsible cells promoting this mechanism [23, 24]. In this study, we investigated concentrations of OPN, RANKL, TNFR1 and TNFR2, known to play a role in mediating osteoclastogenesis, in serum samples from subjects with periodontal disease, rheumatoid arthritis with and without periodontal disease and healthy groups.

Osteoclastogenesis is enhanced in inflammation as many studies have demonstrated the influence of inflammatory cells and mediators such as IL-6 and TNF-α on osteoclast precursors and osteoclast formation [25, 26].

We found that serum RANKL was higher in PD subjects when compared to the healthy group which confirms previous reports [27–29]. RANKL serum levels associated positively with two clinical parameters of high importance regarding disease severity, deep periodontal pockets and MBL. This finding supports findings of a recent study in which RANKL serum levels were also associated with PPD [30]. Besides serum, elevation of RANKL occurs in periodontal soft tissue and gingival crevicular fluid (GCF) in PD patients [31]. Our findings are well aligned with the role of RANKL in mediating osteoclastogenesis in PD. Sources of RANKL in tissues and sera are osteoblasts, B and T cells, dendritic cells, periodontal ligament and gingival fibroblasts [32]. Bone destruction requires critical levels of RANKL for immuno-inflammatory signals to orchestrate accelerated bone loss [33]. High serum concentrations of RANKL in PD and RA (with and without PD) groups were comparable which suggests an extensive involvement of immune and resident cells in periodontal disease. Similarly, increased RANKL/OPG ratio was observed in both PD and RA groups as compared to healthy subjects. With no difference between RA and PD groups, this ratio is considered to tilt the balance of RANKL and OPG towards a bone resorptive status in both groups since high RANKL/OPG ratio influences osteoclast activity. The serum RANKL/OPG ratio is, therefore, a critical factor for determining osteoclast activation [34].

The role of soluble TNF receptors has been suggested as negative modulators of TNF activity. Under normal conditions, serum concentrations of TNF receptors are higher than TNF concentrations. TNFR1 and TNFR2 both inhibit TNF-α, with much greater preferential binding to TNFR1 (177-fold) as compared to that of TNFR2 which is almost 9-fold [35]. Our results showed an inverse correlation between MBL and TNFR1 serum levels in the PD group. This finding is in agreement with previous experimental studies showing antagonizing effects of TNF-α by soluble receptors preventing TNF-mediated bone loss [36]. We also found that serum TNFR1 correlated inversely with PPD≥5mm for all study subjects. This finding can be explained by the fact that deep periodontal pockets form as a result of periodontal tissue attachment loss, mediated by strong pro-inflammatory cytokines such as TNF-α, and allowed to propagate in the absence of physiological TNF inhibitors. Delima *et al* were able to confirm this in their study in which TNF blockers inhibited the loss of connective tissue attachment [37]. TNFR1 concentrations tend to be higher in both serum and synovial fluid of RA patients [38]. The RA with PD group in our study showed higher serum levels of TNFR1 as compared to the PD and RA without PD groups. This may reflect shedding of the receptors in inflamed periodontal tissues in addition to synovial inflammation, thus reflected by higher serum concentrations. Increased levels of both TNF receptors have also been detected in GCF with increasing PPD values [39]. TNFR2 signals cell survival in contrast to TNFR1 which is pro-apoptotic [40]. In autoimmune diseases, it may be due to defects such as polymorphisms in the TNFR2 gene, up regulated expression and receptor shedding.TNFR2 levels were, again, found to be highest in the RA with PD group, decreasing across remaining groups in our study which agrees with previous reports of higher levels of TNFR2 found in serum of patients with rheumatoid arthritis [41]. The differences in serum levels of TNFR1 and TNFR2 could arise due to different shedding mechanisms. Excessive shedding of TNFR2 may be a mechanism aimed at reducing TNF-α activity via TNFR1 thereby indirectly being antagonistic to TNFR1. [42]. Levels of soluble TNFR2 are shown to greatly exceed those of TNFR1 and perhaps reflect a dominant role in down-regulating TNF-α responses [43]. This may be seen as a possible compensatory mechanism to decrease inflammation which might explain the inverse correlation of TNFR2 to TNFR1 in PD and both the RA groups.

Our study showed statistical differences in OPN levels between the PD and RA groups with higher in RA subjects suffering from PD. A possible explanation may be that soluble osteopontin is released by both osteoblasts and osteoclasts which may negatively regulate osteoclast precursor formation as part of a negative feedback mechanism [44]. This mechanism may keep bone resorption in check or play a compensatory role in systemic conditions with osteolysis such as RA. Our findings show that RA subjects with PD have an additional compensatory requirement reflected by higher OPN levels. Another explanation is the role of medication in the RA group as all subjects were being treated with DMARDs. There are studies which have shown a decrease in OPN levels in RA subjects treated with methotrexate [45, 46]. Methotrexate has been shown to reduce serum RANKL levels which have been associated with bone erosion in RA. The marginal bone loss was significantly less in both the RA groups when compared to PD which may be due to DMARDs dampening of inflammatory mechanisms involved in bone loss [47].

Pairwise correlation between markers showed that TNFR1 correlated with all three markers OPN, RANKL, and TNFR2 in RA with and without PD groups while in the PD group, TNFR1 was found to correlate with OPN and TNFR2, but not RANKL. TNFα is not only a potent inducer of bone loss but also an inhibitor of bone formation since it stimulates, via TNFR1, the production of Dickkopf-1 (DKK-1), a secreted glycoprotein which inhibits the Wnt cell signaling pathway which controls tissue regeneration in adult bone marrow [48]. Consequently, up regulation of DKK-1 promotes bone resorption and inhibits bone formation. Another mechanism which reduces bone formation via TNFR1 stimulation is apoptosis of periodontal ligament cells since this can diminish the number of osteoblast precursors available [26]. TNFR1, therefore, plays a salient role in its mediation of increased osseous tissue degradation and retardation of bone repair under sustained inflammatory conditions.

## Conclusion

In our study, we have highlighted the importance of serological osteoclastogenic markers and their modulation due to periodontal disease in subjects with and without RA. Our findings support a new and broader way of recognizing periodontal disease with the likelihood that it is indeed a condition driven/ amplified by mechanisms underlying peripheral osteoclastogenic dysregulation. The role of these markers may prove to be of value in monitoring disease progression and perhaps help evaluate the role of host modulatory drugs in treating periodontal disease.

## Supporting information

S1 Table Data on four assayed proteins for all subjects*. *R = Rheumatoid arthritis (green), P = Periodontal disease (orange), H = Healthy (grey).
(PDF)

S2 Table Data on clinical parameters for all subjects*. *R = Rheumatoid arthritis without PD (green), with PD (dark green), P = Periodontal disease (orange), H = Healthy (grey).
(PDF)
