# Supplementary material for: Periodontal disease influences osteoclastogenic bone markers in subjects with and without rheumatoid arthritis
Source: PLoS One. 2018 Jun 11;13(6):e0197235. doi: 10.1371/journal.pone.0197235 (PMC5995354; doi:10.1371/journal.pone.0197235)
Supplement: S1 Table — *R = Rheumatoid arthritis (green), P = Periodontal disease (orange), H = Healthy (grey). (PDF) [file pone.0197235.s001.pdf]

Subject ID	Ostepontin	RANKL	TNFR1	TNFR2
	pg/mL	pg/mL	pg/mL	pg/mL
R1	997	129	50	4228
R2	792	206	50	2621
R3	1202	139	50	3313
R4	2863	218	402	6571
R5	792	303	998	6263
R6	895	161	50	2257
R7	1615	184	50	2480
R8	1100	242	599	3969
R9	1821	129	312	12637
R10	1305	195	150	4460
R11	1202	195	50	4259
R12	588	184	141	3181
R13	690	184	50	3683
R14	2029	172	744	3938
R15	1202	129	50	3728
R16	588	161	105	7253
R17	7790	407	1245	7683
R18	2863	328	1129	3460
R19	1305	254	203	3653
R20	1202	184	77	4758
R21	1511	206	50	3803
R22	1100	242	345	5760
R23	895	161	50	3563
R24	997	218	114	3386
R25	3073	254	1424	7810
R26	7026	303	1295	7647
R27	4553	502	931	4554
R28	4022	161	1712	4837
R29	8668	407	857	4742
R30	9774	242	1310	8382
R31	8338	433	1502	6726
R32	6268	230	1100	5760
R33	4766	206	614	3758
R34	16737	433	2687	10150
R35	3073	341	990	3504
R36	1305	206	245	13922
R37	1202	266	386	4980
R38	4659	254	1224	4030
P1	942	150	653	5285
P2	1305	242	790	4167
P3	997	230	714	4774
P4	997	195	168	3475
P5	1100	139	50	3181
P6	2237	278	86	3489
P7	1511	161	50	4336
P8	792	474	105	3788
P9	2133	161	691	4695

**P10**	2029	303	279	3239
**P11**	997	254	50	3036
**P12**	1895	328	767	4853
**P13**	739	70	251	3044
**P14**	1305	266	245	3818
**P15**	1360	266	96	5513
**P16**	895	184	50	4045
**P17**	2863	291	552	4321
**P18**	1408	242	505	7076
**P19**	739	230	386	3848
**P20**	739	195	105	1104
**P21**	942	601	254	4679
**P22**	1360	206	1078	6077
**P23**	2029	184	782	4060
**P24**	4608	718	998	3638
**P25**	2863	172	924	5221
**P26**	4234	291	968	5253
**P27**	840	161	245	7503
**P28**	1821	98	50	2964
**P29**	1202	161	50	4336
**P30**	1821	303	50	2820
**P31**	1202	230	105	4585
**P32**	1305	139	50	3534
**P33**	2665	218	337	3954
**P34**	895	242	660	3593
**P35**	3283	206	1410	11585
**P36**	2237	316	706	5628
**P37**	1254	70	144	1456
**P38**	4340	139	2673	14230
H1	1718	184	630	2763
H2	3114	70	50	4908
H3	2004	70	645	4660
H4	3682	70	1620	2880
H5	2665	106	1390	5076
H6	3001	172	1490	3560
H7	6149	70	658	1956
H8	3227	70	1247	3696
H9	3227	70	1218	2604
H10	3797	70	637	1840
H11	6029	70	970	2332
H12	4766	303	794	4964
H13	4234	206	957	3166
H14	7244	206	1408	3475
